# Change of sleep quality from pre- to 3 years post-solid organ transplantation: The Swiss Transplant Cohort Study

**DOI:** 10.1371/journal.pone.0185036

**Published:** 2017-10-11

**Authors:** Hanna Burkhalter, Kris Denhaerynck, Uyen Huynh-Do, Isabelle Binet, Karine Hadaya, Sabina De Geest, for the Psychosocial Interest Group, Swiss Transplant Cohort Study

**Affiliations:** 1 Institute of Nursing Science, Department Public Health, University of Basel, Basel, Switzerland; 2 Center of Sleep Medicine, Hirslanden Group, Zürich, Switzerland; 3 Division of Nephrology and Hypertension, University Hospital of Bern, Bern, Switzerland; 4 Nephrology/Transplantation Medicine, Cantonal Hospital of St.Gallen, St.Gallen, Switzerland; 5 Division of Nephrology and Transplantation, Geneva University Hospital, Geneva, Switzerland; 6 Academic Center for Nursing and Midwifery, Department Public Health, KU Leuven, Belgium; University of California Los Angeles, UNITED STATES

## Abstract

**Background:**

Poor sleep quality (SQ) is common after solid organ transplantation; however, very little is known about its natural history. We assessed the changes in SQ from pre- to 3 years post-transplant in adult heart, kidney, liver and lung recipients included in the prospective nation-wide Swiss Transplant Cohort Study. We explored associations with selected variables in patients suffering persistent poor SQ compared to those with good or variable SQ.

**Methods:**

Adult single organ transplant recipients enrolled in the Swiss Transplant Cohort Study with pre-transplant and at least 3 post-transplant SQ assessment data were included. SQ was self-reported pre-transplant (at listing), then at 6, 12, 24 and 36 months post-transplant. A single SQ item was used to identify poor (0–5) and good sleepers (6–10). Between organ groups, SQ was compared via logistic regression analysis with generalized estimating equations. Within the group reporting persistently poor SQ, we used logistic regression or Kaplan-Meier analysis as appropriate to check for differences in global quality of life and survival.

**Results:**

In a sample of 1173 transplant patients (age: 52.1±13.2 years; 65% males; 66% kidney, 17% liver, 10% lung, 7% heart) transplanted between 2008 and 2012, pre- transplant poor SQ was highest in liver (50%) and heart (49%) recipients. Overall, poor SQ decreased significantly from pre-transplant (38%) to 24 months post-transplant (26%) and remained stable at 3 years (29%). Patients reporting persistently poor SQ had significantly more depressive symptomatology and lower global quality of life.

**Conclusion:**

Because self-reported poor SQ is related to poorer global quality of life, these results emphasize the need for further studies to find suitable treatment options for poor SQ in transplant recipients.

## Background

After a successful transplantation, loss of sleep quality (SQ) typically reduces' recipients' quality of life and may impair survival. However, during follow-up, sleep quality (SQ) self-reports have rarely been collected via standardized protocols. To be feasible, the measurement has to be simple, valid and reliable. Therefore, to differentiate poor from good sleepers, we used a single item from the sleep subscale of the Kidney Disease and Quality of Life–Short Form (KDQoL-SF): “On a scale from 0–10, how would you evaluate your sleep?” Scores of 0–5 were classed as poor; 6–10 indicated good SQ. This item is not yet part of a standardized protocol, and sleep quality is not yet a standardized follow-up criterion.

Poor sleep quality (SQ) is common in hemodialysis patients (49%-53% prevalence) [1, 2]. Kidney transplantation is expected to correct most kidney disease-related abnormalities of the kidney disease and significantly improve the patients’ health. However, a 2011 study reported high (31%) prevalence of poor SQ after kidney transplantation [3]. This may reflect a serious risk regarding patient survival. The Dialysis Outcomes and Practice Patterns Study collected SQ data from 11351 patients in 308 dialysis units across seven countries and reported a 16% higher relative risk of mortality in hemodialysis patients with poor SQ compared to good sleepers [1]. Such a strong link with overall wellbeing and health indicates an equally strong need to detect and understand poor SQ as a post-transplantation health outcome. Patient-reported outcomes (PROs) including SQ are becoming extremely important indicators of the quality of patient care [4]. To date, however, no studies have used PROs (e.g., sleep quality) simultaneously in kidney, heart, liver and lung transplant patients to monitor global quality of life and survival. Data on SQ in kidney, heart, liver and lung transplant patients are scarce, and longitudinal studies are even more so. Therefore, based on data from the Swiss Transplant Cohort Study, the current analysis had three aims: (1) to detect and compare changes in SQ in kidney, heart, liver and lung transplant recipients over time (pre-, 6, 12, 24, and 36 months post-transplant); (2) to compare solid organ transplant recipients groups (kidney, heart, liver and lung) with persistent poor SQ over time; and (3) to compare global quality of life and survival over time (until 3 years post-transplant) in subjects with persistently poor SQ as opposed to those with consistently good and variable SQ.

## Materials and methods

### Design, sample and setting

For this study we used data from the Swiss Transplant Cohort Study, a prospective open cohort study including 1173 patients transplanted in one of the six Swiss transplant centers (Lausanne, Geneva, Basel, Zürich, Bern, St. Gallen). Details of that study are published elsewhere [5]. Kidney, heart, liver and lung transplant recipients were eligible if they were aged 18 years or older, has received a single transplant (i.e., no multiple-organ transplants) enrolled from May 2, 2008 until February 2, 2012, and were followed up until August 11^th^, 2015. After providing written informed consent, organ transplant candidates completed the psychosocial questionnaire (socio-demographic, psychosocial, and behavioral variables, including SQ), pre-transplant (at the time of listing), 6 months post-transplant, 1 year post-transplant, and each year thereafter (described elsewhere) [5, 6]. The Swiss Transplant Cohort Study was approved by the ethics committees overseeing the 6 participating transplant centers. None of the transplant recipients received organs from vulnerable populations, as all organ transplantation is regulated by Swiss law. All donors or next of kin freely provided written informed consent.

### Variables and measurements

#### Sleep quality

*Sleep quality* (SQ) was assessed with a single item derived from the ‘Kidney Disease Quality of Life–Short Form’ instrument, which was initially developed for patients with end-stage renal disease [1]. Patients were asked, “On a scale of 0 to 10 [where 0 represents ‘very bad’ and 10 represents ‘very good’], how would you rate your sleep quality overall?” Based on ROC curve analysis, values below 6 indicated poor SQ [3]. Therefore, we used <6 as a cut-off to define poor SQ [3]. Content validity was good (content validity index (CVI): .81). Concurrent validity (i.e., in relation to the Pittsburgh Sleep Quality Index (r_s_: -.737 p < .01)) and discriminant validity (i.e., versus depression diagnosis and the Depression, Anxiety and Stress scale, and quality of life as measured with the EQ-5D) were good [3]. Predictive validity of the SQ item was demonstrated in the Dialysis Outcome and Practice Pattern Study, [1] as poor SQ predicted mortality.

For our second and third aims, we divided patients into two groups based on the longitudinal pattern of their SQ: those who had persistent poor SQ versus all others (transplant recipients with consistently good or variable sleep quality). As at least two post-transplant measurement points were needed to define patients with persistent poor SQ, inclusion was limited to patients who survived at least 1 year post-transplant.

#### Socio-demographic and clinical variables

Six socio-demographic variables were extracted from the Swiss Transplant Cohort Study database [5, 6]: gender, age in years at the time of transplantation, transplanted organ (kidney, liver, heart, or lung), time between study inclusion and transplantation, highest completed educational level (never completed high school, high school graduate, some college, college graduate) and marital status (single, married/living together, widow/widower, divorced, separated). We also extracted two clinical variables: main immunosuppressive regimen (Azathioprine, Tacrolimus, Cyclosporine, Rapamune, Mycophenolate Acid) and the two most frequent etiologies of the disease pre-transplant (heart transplant: dilated cardiomyopathy and ischemic heart disease; kidney transplant: glomerulonephritis and polycystic kidney disease; liver transplant: hepatitis C and alcohol; lung transplant: chronic obstructive pulmonary disease and cystic fibrosis).

*Depressive symptomatology* was assessed via the 7-item depression subscale of the Hospital Anxiety and Depression Scale, a self-reported non-diagnostic screening instrument. This scale is widely used and well-validated as a screening instrument for depression in the general medical population, and, to some extent, in kidney transplantation contexts [7]. The mean scores for depression in a general German population (N = 4410) were 4.8 (males) and 4.7 (females)[8]. Each item is scored on a scale from 0 to 3, with the total score calculated by summing the seven individual item scores (range: 0–21). A cut-off of ≥8 was used to indicate depression [9]. Sensitivity of the HADS depression subscale using this cut-off was 0.86; specificity was 0.81 for depressive disorder screening [10].

*Global quality of life* was assessed with a visual analogue scale from 0 (worst imaginable quality of life to 100 (best imaginable quality of life). This scale is both widely used and validated for use in German-language settings [6, 11] where it has shown high levels of validity and reliability [12]. The mean score for global quality of life in the general German population was 77.1±17.8 [13]. We used this variable as an outcome as it measures a broad construct of health (a summary of numerous health issues facing transplant recipients) that is close to the transplant recipient’s perspective [12]. *Survival* was defined as the estimated fraction of transplant recipients who would survive over the six years of the study period, it was used as a function to estimate the probability that transplant recipients with poor SQ would eventually differ from those with consistently good or variable SQ after transplantation.

### Statistical analysis

Descriptive analyses of sample characteristics were performed using frequencies, proportions, measures of central tendency (means, medians) and dispersion (standard deviations, interquartile ranges) as appropriate for measurement levels and distributions. Differences in demographic and clinical characteristics among organ groups were screened using Chi-square or Wilcoxon rank-sum tests.

Changes in poor SQ over time for the various organ recipient groups and the overall sample were depicted graphically. They were also modeled via logistic regression analyses using generalized estimating equations to account for the repeated measures [14], and controlling for organ group, age, gender, depressive symptomatology, co-morbidities, immunosuppressive regimen and the time that elapsed between baseline measurement (the pre-transplant psychosocial questionnaire) and transplantation. The model presented was adjusted by retaining only significant covariates.

Two sub-analyses were also performed. The first used an interaction between organ group and time category to generate odds ratio comparisons based entirely on pre-transplant data. The second, performed using post-transplant data, incorporated time as a continuous variable to allow the estimation of linear trends over the post-transplant period. To examine the possible effect of missing follow-up data on model stability, analyses were also performed using a Last-Observation-Carried-Forward (LOCF) imputation strategy.

For our second and third aims, we divided patients into two groups based on their longitudinal sleeping patterns: those who had persistent poor SQ and all others. As at least two post-transplant measurement points were needed to define patients with persistent poor SQ, inclusion was limited to patients who survived at least 1 year post-transplant.

Differences between patients with persistently poor SQ versus those with consistently good or variable SQ were explored via descriptive analyses, and using Chi-square, Wilcoxon rank-sum tests, or Kaplan-Meier estimates as appropriate. We determined both global quality of life and survival via Kaplan Meier analyses, and compared the results with those with consistently good or variable SQ. All analyses were performed using SAS 9.4 (SAS Institute Inc., Cary, NC, USA) software. The alpha level was set at 5%.

## Results

### Sample characteristics overall

**Fig 1:**
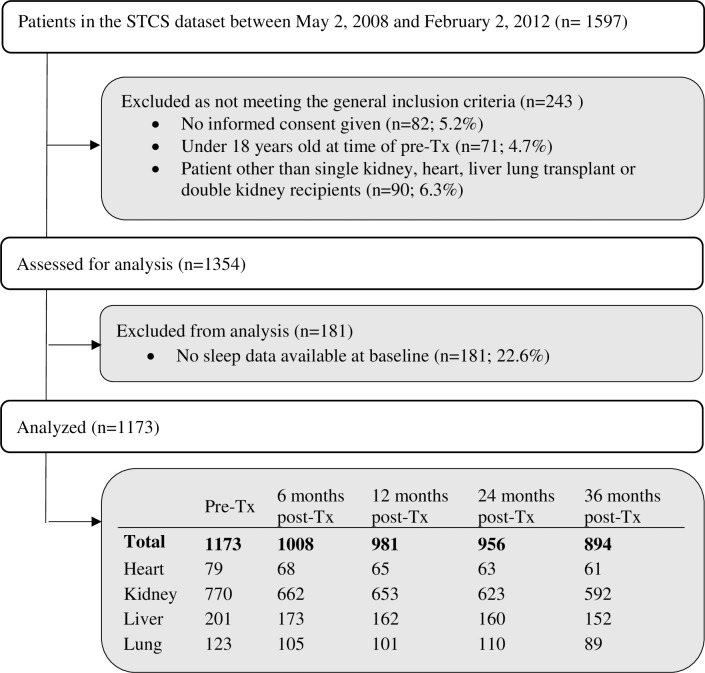
Flow diagram showing the sample as selected from the swiss transplant cohort study overall sample.

As of February 2, 2012, the Swiss transplant cohort study included 1597 patients, of whom 1173 fulfilled the current study’s inclusion criteria: 770 (65%) kidney, 201 (18%) liver, 123 (10%) lung and 79 (7%) heart transplant candidates. Fig 1 depicts the overall sample size and the number of included respondents per organ group at each data collection point. Demographics and clinical characteristics of the 1173 transplant recipients included in the analyses are provided in Table 1. At pre-transplant measurement, the average patient age was 50.1±13.2 years; almost two-thirds (65.5%) were male; and most (66.1%) were married or living with a partner. The median period between pre-transplant assessment and transplantation was 7.7±9.6 months.

**Table 1:** Sample characteristics at pre-transplant for all organs and per organ group.

		**Pre-Tx All**	**Heart**	**Kidney**	**Liver**	**Lung**	**P-value**
**Assessed at listing**		N = 1173 (100%)	N = 79 (6.73%)	N = 770 (65.64%)	N = 201 (17.14%)	N = 123 (10.49%)	
Age	Mean (std) in years (Age range 18–79 years)	52.06 ±13.21	50.66 ±12.2	52.13 ±13.74	53.65 ±10.86	49.91 ±13.68	0.16
Gender	Male–N (%)	768 (65.47)	64 (81.01)	513 (66.62)	132 (65.67)	59 (47.97)	< .0001
Living situation	Divorced–N (%)	110 (9.47)	10 (12.82)	61 (8.02)	23 (11.56)	16 (13.01)	0.098
	Married/living together–N (%)	768 (66.15)	45 (57.69)	510 (67.02)	138 (69.35)	75 (60.98)	
	Separated–N (%)	35 (3.01)	5 (6.41)	18 (2.37)	7 (3.52)	5 (4.07)	
	Single–N (%)	219 (18.86)	15 (19.23)	148 (19.45)	30 (15.08)	26 (21.14)	
	Widow/widower–N (%)	29 (2.5)	3 (3.85)	24 (3.15)	1 (0.5)	1 (0.81)	
	Missing = 12						
Highest completed educational degree	Never completed high school N (%)	807 (69.15)	55 (69.62)	531 (69.41)	135 (67.5)	86 (69.92)	0.961
	High school graduate N (%)	73 (6.25)	7 (8.86)	45 (5.88)	15 (7.5)	6 (4.88)	
	Some college N (%)	177 (15.17)	12 (15.19)	114 (14.9)	31 (15.5)	20 (16.26)	
	College graduate N (%)	110 (9.42)	5 (6.33)	75 (9.8)	19 (9.5)	11 (8.94)	
	Missing = 6						
Depressive	Median (25^th^; 75^th^ Percentile)	4 (2; 7)	5 (3; 8.17)	4 (2; 6)	4 (2; 7)	5 (3; 8)	< .0001
symptomatology	Missing = 20						
Global quality of life	Mean (std) range 0–100 Missings = 55	55.82±22.50	43.47 ±17.53	59.98 ±21.70	55.26 ±23.69	38.77 ±16.70	< .0001
Time between inclusion in STCS and Tx	Mean (std) in years	-7.96 ±9.58	-5.96 ±6.7	-8.92 ±10.76	-4.92 ±5.64	-8.26 ±6.83	0.0002
		**Pre-Tx All**	**Heart**	**Kidney**	**Liver**	**Lung**	**P-value**
**Assessed at Tx Time**							
Comorbidities	History of cancer–N (%)	224 (19.1)	11 (13.92)	111 (14.42)	83 (41.29)	19 (15.45)	< .0001
	Missing = 0						
	History of Cardiopulmonary diseases–N (%)	662 (56.44)	79 (100)	393 (51.24)	67 (33.33)	123 (100)	0.043
	Missing = 3						
	History of metabolic, endocrine or kidney diseases–N (%)	1054 (89.85)	66 (83.54)	770 (100)	142 (70.65)	76 (61.79)	< .0001
	Missing = 0						
	History of skin cancer–N (%)	62 (5.28)	3 (3.8)	45 (5.85)	6 (2.99)	8 (6.5)	0.93
	Missing = 1						
Nr. Comorbidities at time of Tx	Median (25^th^; 75^th^ Percentile)	2 (1; 2)	2 (2; 2)	2 (1; 2)	1 (1; 2)	2 (1; 2)	<0.0001
	Missing = 0						
IS at time of Tx	Azathioprine-Tacrolimus	32 (2.74)	20 (25.32)	8 (1.04)	3 (1.52)	1 (0.81)	
(Percentage based on	Cyclosporine- Mycophenolate A	270 (23.16)	43 (54.43)	116 (15.14)	36 (18.18)	75 (60.98)	
Organ group)	Cyclosporine- EC-Mycophenolate A	66 (5.66)	2 (2.53)	61 (7.96)	1 (0.51)	2 (1.63)	
	Tacrolimus	745 (63.83)	10 (12.66)	555 (72.45)	135 (68.18)	45 (36.59)	
	Other immunosuppressiva	45 (3.86)	4 (5.06)	20 (2.61)	21 (10.61)	0 (0)	
	Rapamune—Mycophenolate Acid	8 (0.69)	0 (0)	6 (0.78)	2 (1.01)	0 (0)	
	Total	1166 (100)	79 (6.78)	766 (65.69)	198 (16.98)	123 (10.55)	0.381
	Missing = 7						
IS at 6 Months	Azathioprine-Tacrolimus	28 (2.8)	3 (4.41)	19 (2.89)	4 (2.35)	2 (1.9)	
(Percentage based on	Cyclosporine- Mycophenolate A	189 (18.89)	24 (35.29)	80 (12.16)	28 (16.47)	57 (54.29)	
Organ group)	Cyclosporine- EC-Mycophenolate A	49 (4.89)	2 (2.94)	39 (5.93)	2 (1.18)	6 (5.71)	
	Tacrolimus	664 (66.34)	29 (42.65)	492 (74.77)	105 (61.76)	38 (36.19)	
	Other immunosuppressiva	52 (5.19)	6 (8.82)	16 (2.43)	28 (16.47)	2 (1.9)	
	Rapamune—Mycophenolate Acid	19 (1.9)	4 (5.88)	12 (1.82)	3 (1.76)	0 (0)	
	Total	1001 (100)	68 (6.79)	658 (65.73)	170 (16.98)	105 (10.49)	<0.0001
	Missing = 6						

**Legend:** Tx = transplantation; std = standard deviation; STCS (Swiss Transplant Cohort Study); AZA = Azathioprine; TAC = Tacrolimus; CSA = Cyclosporine; Rap = Rapamune; MPA = Mycophenolate Acid; MPA-EC = Enteric-coated Mycophenolate Acid; IS = Immunosuppression; COPD = Chronic Obstructive Pulmonary Disease

The follow-up time ranged from 18 months to 7.1 years, with a median of 4.1 years (interquartile range 2.0). Of the 1173 patients included in the study at the pre-transplant measurement, 894 (76.2%) had available data over 36 months. One hundred, eight (9.2%) patients had died by the end of the study.

Of the 765 kidney insufficient patients, 16% (N = 119) were transplanted pre-emptively (estimated GFR (eGFR) was 10 ml/min/1.73m^2^), 69% (N = 531) were on hemodialysis and 15% (N = 115) on peritoneal dialysis. The mean time on dialysis was 4.0±5.0 years. The eGFR at 36 months was 55 ml/min/1.73m^2^ post-kidney transplantation and 72 ml/min/1.73m^2^ post-liver transplantation. The pre-liver-transplantation MELD score was 20±9 and the pre-heart-transplantation Ejection Fraction was 24.4±13.0%.

### Changes of poor sleep quality between organ groups over time

**Fig 2:**
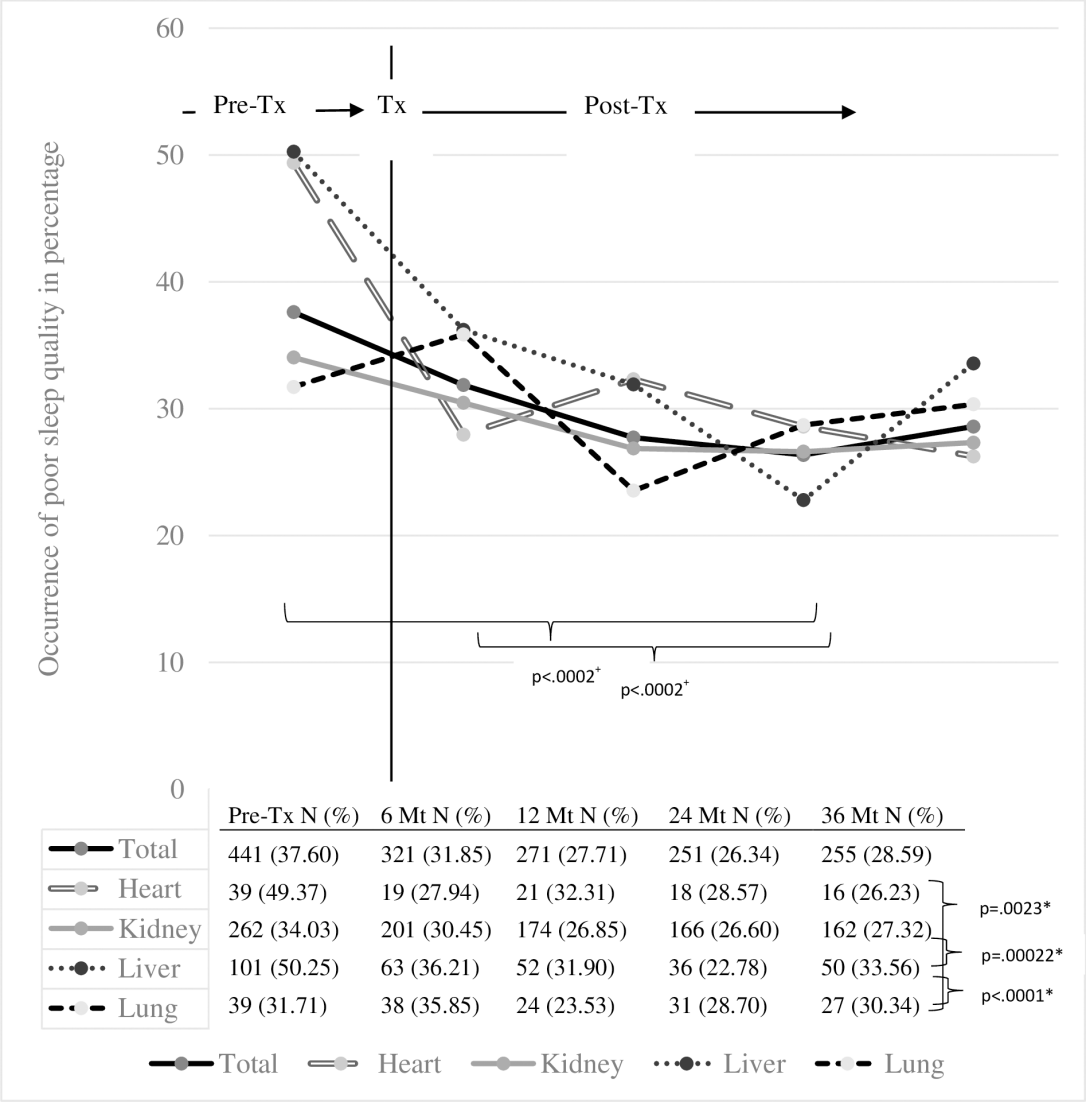
Change in poor SQ from pre-transplant to 3 years post-transplant (overall & per transplant group).

Fig 2 shows the percentages of patients with poor SQ at each data collection point. Pre-transplant, 38% of the sample reported poor SQ. At 24 months post-transplant, poor SQ was reported at its lowest level (26%). Fewer heart, kidney and liver transplant recipients reported poor SQ at six months compared to pre-transplant (Table 2). For the kidney and liver transplant groups, this improvement continued until the 1-year measurement. Overall, as illustrated in Table 3, patients’ odds of poor SQ were higher at baseline (OR 1.45; 95%CI: 1.21–1.74) and 6 months post-transplant (OR 1.44; 95%CI: 1.21–1.72) compared to 24 months post-transplant.

**Table 2:** Modeling of poor SQ over time and across different organ transplant groups.

	Contrast	Odds ratio (95% confidence interval)	Chi^2^ value	p-value
Intercept		0.12 (0.09;0.17)	164.7	< .0001
Male Gender		1.44 (1.18;1.77)	13.2	0.0003
Depressive symptomatology ^+^		1.23 (1.20;1.25)	297.46	< .0002
Measurement Point	Baseline vs 24 months post-Tx (*)	1.45 (1.21;1.74)	16.73	< .0002
	6 months post-Tx vs 24 months post-Tx	1.44 (1.21;1.72)	17.21	< .0002
	12 months post-Tx vs 24 months post-Tx	1.19 (1.00;1.42)	3.8	0.0512
	36 months post-Tx vs 24 months post-Tx	1.13 (0.96;1.33)	2.18	0.14
	Kidney vs Liver	0.74 (0.59;0.94)	6.18	0.0129
	Lung vs Liver	0.73 (0.50;1.06)	2.81	0.0939
	Heart vs Liver	1.04 (0.66;1.63)	0.03	0.874
	Lung vs Kidney	0.98 (0.71;1.37)	0.01	0.9135
	Kidney vs Heart	0.72 (0.47;1.09)	2.45	0.1176
	Heart vs Lung	1.42 (0.85;2.37)	1.83	0.1756

**Explanation**: Logistic regression model predicting poor SQ (N = 1173). We controlled for organ group, age, gender, depressive symptomatology, co-morbidities, immunosuppressive regimen and the time that elapsed between baseline measurement and transplantation. Only significant confounders were retained in the final model.

**Legend**: SQ: Sleep quality; Tx: transplantation; STCS: Swiss Transplant Cohort Study; + = Variable used as a continuous and time dependent variable

(*) Odds ratio can be interpreted as the adjusted odds of poor SQ at enrolment compared to the reference category of poor SQ at 24 months.

**Table 3:** Differences in poor SQ over time between organ transplant groups pre-Tx.

Contrast	Odds ratio (95% confidence interval)	Chi^2^ value	p-value
Heart vs Kidney	0.27 (-1.13;-0.06)	4.69	0.0303
Liver vs Kidney	0.17 (-0.97;-0.3)	14.15	0.0002
Lung vs Kidney	0.23 (-0.01;0.9)	3.7	0.0543
Heart vs Liver	0.3 (-0.54;0.63)	0.02	0.8818
Heart vs Lung	0.34 (-1.71;-0.37)	9.34	0.0022
Liver vs Lung	0.26 (-1.6;-0.57)	17.01	< .0001

**Explanation**: Model of Table 2 (controlled for gender and depressive symptomatology), with an added interaction between organ groups and time categories. Only contrasts between organ groups are reported.

Pre-transplant liver patients reported the highest occurrence of poor SQ (50%), followed closely by heart transplant candidates (49%). Both these groups differed significantly from their lung transplant (32%) and kidney transplant (34%) counterparts (Table 3). A decrease in poor SQ among kidney (OR 0.91; 95%CI: 0.84–0.97; p = 0.005; Table 4) and liver transplant recipients (OR 0.80; 95%CI: 0.69–0.93; p = 0.004) could be observed via linear modeling of post-transplant developments (Table 4). Other organ recipient groups showed no statistically significant changes over time. In terms of estimates and inferences, replacing missing values by imputing the last observed values yearly until the end of the observation period only moderately (not statistically significant) affected the results in any of the tables.

**Table 4:** Poor SQ over time (in years) with interactions across different organ transplant groups.

Contrast	Odds ratio (95% confidence interval)	Chi^2^ value	p-value
Heart	0.84 (0.69;1.04)	2.5	0.1139
Kidney	0.91 (0.84;0.97)	7.79	0.0052
Liver	0.80 (0.69;0.93)	8.45	0.0037
Lung	1.03 (0.86;1.24)	0.13	0.7164

**Explanation**: Logistic regression model predicting poor SQ (N = 1173), controlling for gender and depressive symptomatology and including an interaction between organ groups and continuous time. Because of nonlinearities between pre- and post-transplant time-points, only post-transplant data were included. Odds rations represent regression slopes over time for the different organs.

### Sample characteristics and correlates for transplant recipients with persistent poor SQ

Table 5 shows the characteristics of the overall sample at 6 months post-transplant, alongside those of persistent poor SQ and of all others (those with consistently good or variable SQ). The persistently poor SQ group accounted for 10% of the sample (N = 107; 10.4%). Poor SQ was more prevalent in patients with lower education (p = 0.03) and in those with more depressive symptoms (p = < .0001).

**Table 5:** Sample characteristics at pre-transplant overall and per sleep quality pattern.

		Overall	Persistent poor SQ	Consistently good or variable SQ	p
N (%)		1136 (100)	107 (10.4)	1029 (90.6)	
Age	Mean (std) in years (Age range 18–79 years)	52.10 ±13.23	49.98 ±10.86	52.32 ±13.44	**0.02**
Gender	Male–N (%)	748 (65.85)	60 (56.07)	688 (66.86)	0.0664
Living situation	Married/living together–N (%)	750 (66.61)	63 (60.00)	687 (67.29)	0.0449
Highest completed educational level	No completed high school N (%)	779 (68.88)	86 (80.37)	693 (67.68)	**0.0312**
	High school graduate N (%)	72 (6.37)	5 (4.67)	67 (6.54)	
	Some college N (%)	173 (15.30)	7 (6.54)	166 (16.21)	
	College graduate N (%)	107 (9.46)	9 (8.41)	98 (9.58)	
Time between inclusion in STCS and Tx	Mean (std) in years	7.89 ±9.49	7.08 ±8.53	7.98 ±9.58	0.62
Depressive symptomatology	Median (25^th^; 75^th^ Percentile)	4 (2; 7)	7 (4; 11)	4 (2; 7)	**< .0001**
Nr. Comorbidities	Median (25^th^; 75^th^ Percentile)	2 (1; 2)	2 (1; 2)	2 (1; 2)	0.9900
Global quality of life pre-Tx	Mean (std) range 0–100	55.89±22.56	42.19±21.01	57.31±22.25	**< .0001**
Global quality of life post-Tx	Mean (std) range 0–100	74.34±17.67	59.69±21.51	75.63 ±16.68	**0.0001**
Survival	Probability at max 6 years post-Tx	0.83	0.79	0.83	0.6154

**Explanation:** Patients were divided into two groups based on their longitudinal sleeping patterns: those who had persistent poor SQ and all others. Differences were explored between these 2 groups.

### Global quality of life and Survival for Patients with persistent poor SQ

Pre-transplant, the global quality of life rating was 55.9±22.6; 6 months post-transplant, it increased to 74.3±17.7. Graft recipients with persistently poor SQ over time had a significantly lower mean global quality of life (59.7±21.5; p = < .0001) compared to those with consistently good or variable SQ. This result was consistent when controlling for organ group, age, gender, comorbidities and time between inclusion in the Swiss Transplant Cohort Study and transplantation. Missing data over time did not change the results (as determined by a sensitivity analysis using a last observation carried forward imputation method). At 6 years post-transplant, survival probability was 0.83 for those with consistently good or variable SQ and 0.79 for patients with persistent poor SQ (not significant).

## Discussion

To the best of our knowledge, the current work represents the largest study to date to simultaneously investigate self-reported SQ in heart, lung, liver and kidney transplant groups using a single protocol. We found that poor SQ affected roughly one-third of transplant recipients throughout the measurement period, but generally improved from pre-transplant until 1 year post-transplant. Significant differences in perceived SQ were observed between organ groups. As reported in previous studies, poor SQ is common pre- and post-transplant; however, those studies were limited regarding follow-up periods and sample sizes, and used various SQ assessment methods.

### Changes in poor sleep quality over time

The range of poor SQ measured with the Pittsburgh Sleep Quality index is extremely wide in the published studies: 28–69% in heart [15], 8–62% in kidney [3, 15–21] [22], 51–72% in liver [15, 23, 24] and 32–54% in lung transplant recipients [15, 25]. Measured with the Pittsburgh Sleep Quality Index the prevalence of poor SQ in the general population ranges from 22% [26] to 45% [27]. As there are no prevalence data of poor SQ assessed with the single SQ item we cannot conclude that our prevalence is higher than in the general population. The most appropriate conclusion drawn from these results is the comparison of the prevalence of poor SQ across organ transplant groups and over time.

The improvement indicated from pre- to post-kidney transplantation confirms the findings of previous studies [21]. For example, one recent study showed that 46% of kidney transplant recipients experienced a clinically relevant improvement in overall sleep quality, while 21% experienced a clinically relevant deterioration [21] over 5 years of follow up. Over time, kidney and liver transplant recipients showed a significant decrease in poor SQ. The drop might be connected with the normalization of life with a transplanted organ and return to work [28]. Compared to heart and lung groups [29], the fear of rejection and the frequency of follow-up visits are normally reduced at 36 months post-kidney and liver transplantation. Liaveri et.al.’s study in renal transplant recipients reported improved quality of life after transplantation; however this benefit does not seem to extend to sleep quality [30]. In that case, sleep quality was affected by frequency of post-traumatic stress symptoms, depression, restless leg syndrome, high diastolic blood pressure, and pain [30].

One hypothesis to explain these connections is that fatigue (reported prevalence post-kidney transplantation: 40%-50% [31]) interferes with perceived sleep quality. It also limits a person's ability to carry out ordinary daily activities, with a bidirectional impact on sleep [32]. Daytime sleepiness improves [22] after kidney transplantation; however, those for whom it persists are more prone to immunosuppression non-adherence [33]. No [22] or little improvement [34] was found with objective measurements such as melatonin secretion or circadian rhythms.

No significant change over time was found in heart and lung transplant recipients. Their slope showed fluctuation that could represent the health instability in these transplant recipients. In a qualitative study the recipients’ uncertainty reflected their complex medication regimens, unpredictable future health/prognosis, and complex role and identity challenges [35]. Heart and lung transplant recipients’ medication regimen may also be more complex than those of liver and kidney recipients [36, 37].

### Characteristics of transplant recipients with persistent poor SQ over time

Transplant recipients with persistent poor SQ (10% of the sample) need to consult with a sleep medicine expert. Occasionally, not getting enough sleep is completely normal; however, over months and years, poor SQ results in a suffering trajectory. E.g., we found that transplant recipients with persistent poor SQ score significantly higher on the depressive symptomatology scale. This association mainly corroborates previous findings [38, 39] in transplant recipients. Among patients with lower educational levels and more depressive symptomatology, the predisposition for poor SQ has previously been confirmed by Patel et. al. [40] and in a review by Tsuno et.al. [41].

Transplant recipients whose poor SQ persisted over extended periods had a significantly lower mean global quality of life compared to those with consistently good or variable sleep quality. A study including patients suffering from chronic illnesses such as coronary artery disease showed that both sleep quality and sleep quantity impact these patients´ quality of life [42]. This is congruent with a recent review´s finding that pronounced persistent poor sleep is a major risk for psychiatric, cardiovascular, metabolic or hormonal co-morbidity and mortality [43].

We found no increased mortality risk among transplant recipients whose poor SQ persisted over time. Poor SQ, depression and poor global quality of life commonly co-occur in transplant recipients in ways that suggest bi-directional influence. Evidence that poor SQ is part of a cluster of symptoms has been found in cancer patients [44]. However, further studies are needed as a recent study (N = 152 renal transplant recipients) reported an amelioration of renal function post-transplantation that improved several aspects of quality of life, but with no beneficial effect on self-reported sleep [30]. Finally, as the high prevalence of poor SQ is an increasing public health issue [45], its associations with poorer outcomes indicate a need for regular assessment.

### Clinical relevance and possible interventions

Of the sleep assessment methods currently available, self-reporting is the most accessible, least expensive and simplest to integrate into daily clinical practice [1, 3]. Our analysis showed no association between poor SQ and lower survival; however, the dialysis Outcome and Practice Patterns Study associated poor SQ with higher mortality [1]. Therefore, it is worth integrating this simple question into standard follow-up care while monitoring other parameters.

If during the follow-up visit a score >6 on the single-item self-report of SQ is given, a more in-depth questionnaire or a sleep assessment-asking among other things, about sleep aids should follow. Transplant recipients are educated not to take over-the-counter medicines or herbal supplements without first discussing them with the transplant clinician [46]. In the general population, use of over-the-counter sleep aids (e.g., herbal pills, melatonin or antihystamines) is common [47]. However herbal formulations with an anxiolytic and sedative effect might precipitate liver failure [48]. Even in healthy people, sleep aids are always potentially toxic to the kidneys and liver [49], with common adverse effects including daytime somnolence or decreased alertness [50]. Additional risks have to be acknowledged for all transplant patients. To deal with the problem of persistent poor sleep quality, though, it is crucial to know which factors or combinations of factors influence patients’ perceptions of their SQ. To understand these factors and move forward, system-level improvement is needed in transplant recipient follow-up care.

### Strength and limitation

A limitation of the Swiss Transplant Cohort Study, is that, in the interests of their instrument´s overall feasibility, its designers could include only a very short and quick scale on SQ. Compared to a well-established tool such as the Pittsburgh Sleep Quality Index, our single-item solution is a weak measurement of sleep quality. As a counterpoint, the Swiss Transplant Cohort Study includes a large sample of solid organ transplant recipients reporting sleep quality issues over time.

One further weakness is that the Swiss Transplant Cohort Study psychosocial questionnaire does not permit investigation of specific sleep disorders such as restless leg syndrome, periodic limb movement, or sleep apnea. However this single-item scale´s validity has been confirmed in kidney studies, suggesting further uses in the clinical context of organ transplantation [1–3]. This single-item SQ measurement could easily be incorporated in ambulatory clinic follow-up care for transplant recipients.

## Conclusion

Sleep quality improved following transplantation; but poor sleep quality was prevalent in kidney, heart, liver and lung transplanted patients. Therefore, SQ should be assessed routinely in solid organ transplant recipients.

## Abbreviations

SQ: Sleep Quality
STCS: Swiss Transplant Cohort Study
